# Early Detection for Cases of Enterovirus- and Influenza-Like Illness through a Newly Established School-Based Syndromic Surveillance System in Taipei, January 2010 ~ August 2011

**DOI:** 10.1371/journal.pone.0122865

**Published:** 2015-04-15

**Authors:** Ting Chia Weng, Ta Chien Chan, Hsien Tang Lin, Chia Kun Jasper Chang, Wen Wen Wang, Zheng Rong Tiger Li, Hao-Yuan Cheng, Yu-Roo Chu, Allen Wen-Hsiang Chiu, Muh-Yong Yen, Chwan-Chuen King

**Affiliations:** 1 Department of Medicine, College of Medicine, National Taiwan University (NTU), Taipei, Taiwan, Republic of China; 2 Research Center for Humanities and Social Sciences, Academia Sinica, Taipei, Taiwan, R.O.C; 3 Department of Health, Taipei City Government, Taipei, Taiwan, R.O.C; 4 Institute of Epidemiology and Preventive Medicine, College of Public Health, NTU, Taipei, Taiwan, R.O.C; 5 Department of Pediatrics, NTU Hospital, Taipei, Taiwan, R.O.C; 6 Taipei City Hospital, Department of Health, Taipei City Government, Taipei, Taiwan, R.O.C; 7 School of Medicine, National Yang-Ming University, Taipei, Taiwan, R.O.C; Alberta Provincial Laboratory for Public Health/ University of Alberta, CANADA

## Abstract

School children may transmit pathogens with cluster cases occurring on campuses and in families. In response to the 2009 influenza A (H1N1) pandemic, Taipei City Government officials developed a School-based Infectious Disease Syndromic Surveillance System (SID-SSS). Teachers and nurses from preschools to universities in all 12 districts within Taipei are required to daily report cases of symptomatic children or sick leave requests through the SID-SSS. The pre-diagnosis at schools is submitted firstly as common pediatric disease syndrome-groups and re-submitted after confirmation by physicians. We retrieved these data from January 2010 to August 2011 for spatio-temporal analysis and evaluated the temporal trends with cases obtained from both the Emergency Department-based Syndromic Surveillance System (ED-SSS) and the Longitudinal Health Insurance Database 2005 (LHID2005). Through the SID-SSS, enterovirus-like illness (EVI) and influenza-like illness (ILI) were the two most reported syndrome groups (77.6% and 15.8% among a total of 19,334 cases, respectively). The pre-diagnosis judgments made by school teachers and nurses showed high consistency with physicians’ clinical diagnoses for EVI (97.8%) and ILI (98.9%). Most importantly, the SID-SSS had better timeliness with earlier peaks of EVI and ILI than those in the ED-SSS. Furthermore, both of the syndrome groups in these two surveillance systems had the best correlation reaching 0.98 and 0.95, respectively (p<0.01). Spatio-temporal analysis observed the patterns of EVI and ILI both diffuse from the northern suburban districts to central Taipei, with ILI spreading faster. This novel system can identify early suspected cases of two important pediatric infections occurring at schools, and clusters from schools/families. It was also cost-effective (95.5% of the operation cost reduced and 59.7% processing time saved). The timely surveillance of mild EVI and ILI cases integrated with spatial analysis may help public health decision-makers with where to target for enhancing surveillance and prevention measures to minimize severe cases.

## Introduction

Adequate community-based infectious disease surveillance can provide advanced awareness of emerging threats and promote timely, evidence-based prevention and control strategies [1]. Children infected with enterovirus exhibit from asymptomatic, mild symptoms (enterovirus-like illness, EVI) to severe forms with neurologic sequelae, cardiopulmonary failure or death [2, 3]. With no effective antiviral therapy available and an EV71 vaccine under development [4–7], surveillance of EVI is necessary to detect early signals, reduce cluster size, and slow spreading. School children infected with influenza have higher viral loads, longer shedding of viruses, and higher attack rates, thus are more likely to amplify transmission and create clusters of influenza-like illness (ILI) cases in families and schools [8–10]. An effective early outbreak detection system for school children is, therefore, essential to public health [11, 12].

To identify cases or clusters of infectious diseases that require an immediate public health response, we developed a nationwide Emergency Department based Syndromic Surveillance System (ED-SSS) in Taiwan after the outbreak of severe acute respiratory syndrome (SARS) in 2003 [13]. During the 2009 pandemic influenza H1N1 (pdm H1N1/09), the numbers of student ILI cases in Taipei surged**.** A community-based internet accessible, timely syndromic surveillance system thus replaced past facsimile-based paper reporting. Taipei City had a population of 425,709 schoolchildren from preschools to secondary schools (16% of total) in an area of 271.80 km^2^ in 2010 [14] and higher isolation rates of the novel pdm H1N1/09 virus in Taiwan [15]. Therefore, the Department of Health in Taipei (Taipei-DOH) implemented the first daily School-based Infectious Disease Syndromic Surveillance System (SID-SSS) with a 100% coverage rate of registered educational institutions from preschools to colleges and universities beginning in January 2010.

This is the first report to demonstrate the results of a newly-developed syndromic surveillance system with cases identified by non-medical professionals for early detection of common pediatric infectious diseases in the community. The specific aims of this study were: (1) to evaluate the timeliness and feasibility of the novel SID-SSS routinely operating in schools, (2) to find ways to improve its application in public health, and (3) to share our valuable experiences in Taipei with public health professionals in other countries to promote better global surveillance networks in the future.

## Methods

### System Design

**Fig 1:**
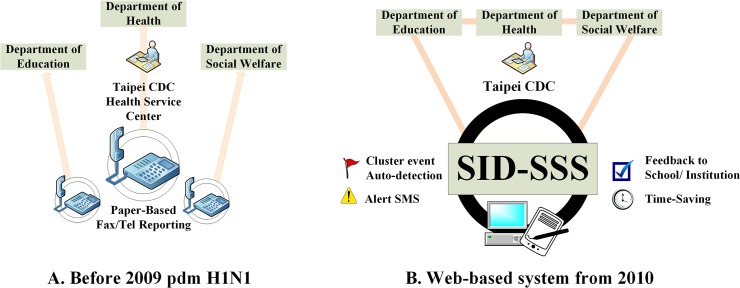
The past (A) and current (B) syndromic surveillance systems of infectious diseases used in Taipei schools and other educational institutions. (A) Traditional paper-based infectious disease reporting system through Fax submission (till 31 December, 2009) (B) Newly developed School-based Infectious Disease Syndromic Surveillance System (SID-SSS) starting from 1 January, 2010. **Taipei CDC:** Centers for Disease Control in Taipei.

The Taipei-DOH invited the other two departments (Education and Social Welfare) to design a more efficient and collaborative infectious disease surveillance system, SID-SSS (http://subweb.health.gov.tw/infection/), by initially adapting electronic-reporting software with a data-sharing user interface. The collected real-time data have auto-feedback which links to both these three government agencies and the participating schools and institutions. Once a cluster of cases is detected, epidemiological investigation and other public health prevention and control measures could be quickly initiated to minimize the health threat. Fig 1 illustrates the flow diagrams of change from a paper-based to a web-based infectious disease reporting system in Taipei’s school surveillance.

### Registered Institutions

School nurses and class teachers of all registered schools and educational institutions located in the 12 districts of Taipei City are the first line for surveillance. The syndrome groups of students’ illness and their absence due to common pediatric infectious diseases should be reported to the SID-SSS daily. Totally, there were 1072 preschools (age-group of 0–6 year-olds, including daycare, nursery centers and kindergartens), 148 primary schools (7-12-year-olds), 133 secondary schools (13-18-year-olds), 29 universities/colleges/professional schools, and 2279 after-school centers.

### Data Collections and Responses

The reporting focuses on the five syndrome groups related to the most common pediatric infectious diseases in Taiwan: (1) enterovirus, (2) influenza, (3) conjunctivitis, (4) diarrhea, and (5) others (chicken pox, scabies, and head louse infection). The system requires all school nurses or class teachers (for those preschools without nurses) of every enrolled institution to collect the following two types of data on a daily basis: (1) obligatory reporting of clinical and epidemiologic information (type of the suspected infection, date of symptom onset, and the class, grade, gender and age of the sick student), and (2) optional supplementary information recorded with a text format (total numbers of absent days with sick children, family members with similar symptoms, medical care transfers, physician diagnosis, and school case management). From this, the SID-SSS can automatically detect the presence and numbers of syndromic cases and school clusters, and capture the secondary cases in schools and from families as well.

A “cluster” was defined as two or more epidemiologically-linked cases (at the same class in the same school) occurring within seven consecutive days for EVI, and those within three consecutive days for ILI, red eye, diarrhea and others. Once a cluster happens, a real-time text-message alert through short message service (SMS) is immediately sent to the public health professionals for further epidemiologic investigations. Then, an epidemiology team in charge of daily monitoring for the disease trends and the Centers for Disease Control in Taipei (e.g. Taipei-CDC) decides the follow-up public health risk management. Different diseases have various criteria to trigger subsequent investigations. The team initiated outbreak investigations and control measures, once the index EVI case, or more than 2 EVI cases, would be detected within a one-time interval, or an EVI cluster event occurred during an epidemic season. The investigation of ILI cases started when the numbers of ILI cases showed abnormally high after comparing the trends of ILI cases in the same weeks in previous years as well as ILI school or family clusters.

To obtain better data from schools, every submitted case was categorized based on syndrome reports rather than absenteeism. Routinely, symptomatic students detected in classes and the families were telephone-interviewed by teachers to find out whether their symptoms belonged to any of the aforementioned five categories. This information and the verifying symptoms of sick children who sought medical aid at a school’s health rooms/centers were all compiled and finally reported to the SID-SSS through school nurses/teachers.

In the web-reporting process, school nurses or teachers first selected one of the infectious diseases’ syndrome groups from the five categories (“red eyes” on website for conjunctivitis) and then reported related symptoms and signs of the chosen disease from the next window (such as options of herpangina, hand-foot-mouth diseases and others for EVI). In Taipei, nearly 100% of primary and secondary schools had at least one registered nurse on-site in the compulsory education system, because each school is required to have at least one or two registered nurse(s) for schools with a total number of classes fewer than 40 or with over 40 classes, respectively. Several training workshops were held in December, 2009 to teach school nurses and teachers the public health significance, the differential diagnosis of common pediatric diseases, operation procedures to report to the SID-SSS**,** legal requirements for reporting**,** and possible penalties for non-reporting. In addition, post-operation feedback workshops were also held to promote the SID-SSS, and to identify users’ difficulties for further improvement.

### Data Analysis and Evaluation

We retrieved the data from 1 January, 2010 to 31 August, 2011 covering all 12 districts of Taipei City for evaluating the quality, temporal trends and timeliness of these data. For the quality of the data, the SID-SSS reserves a column to be filled out the subsequent results of physicians’ diagnoses. Therefore, teachers and nurses can compare the consistency between their initial judgments and the follow-up clinical diagnoses from physicians if the school children seek medical care.

To determine the timeliness of the SID-SSS, we compared the weekly data of the four most reported syndrome groups (EVI, ILI, red eye and diarrhea), with those from the nationwide hospital-based ED-SSS according to the diagnostic codes described in the International Classification of Diseases, Clinical Modification, 9^th^ edition [13]. Statistical analysis was performed using SPSS version 16.0 (*SPSS*, Inc, *Chicago*, *IL*, USA). Furthermore, the National Health Insurance Research Database (NHIRD) with over 99% coverage of approximately 25.68 million Taiwan citizens was also used as the second external dataset. To better evaluate the SID-SSS, we applied the Longitudinal Health Insurance Database 2005 (LHID2005), a database randomly sampled from the year 2005 registry for beneficiaries, showed no significant differences in the distributions of gender, age from the original NHIRD [16]. Cases (clinic visits) were defined by the selected ICD-9 codes, and health seeking population was estimated by a capture-recapture approach [17, 18]. Chi-square tests and the Pearson correlation coefficients were used for percentage comparisons and correlations, respectively. T-tests were used to compare the means of incidence obtained from different school groups (preschool, primary, and secondary school students and others). The statistical analysis was performed using SAS 9.1.3 (SAS Institute, Cary, NC, U.S.A.). Variables with p-value less than 0.05 were considered statistically significant.

### Spatio-temporal Analysis

To investigate the spreading pattern across Taipei, we selected the 10 weeks of the peak periods of EVI (Week 19 to 28, 2010) and ILI (Week 47, 2010 to Week 4, 2011). All 12 districts were classified into the four geographic groups—A, B, C, and D, based on their spatial relationships and socioeconomic development status [19]. Group A, B, C and D represented the Taipei Central Businesses District (Taipei-CBD), the para-CBD, the north, and south-east suburban areas, respectively. The district-specific incidence rates of ILI or EVI syndromic cases per 1000 students were calculated by the number of the reported cases for each district divided by the total number of children from the registered preschools to secondary schools in that district. These data were ranked as 1^st^ to 5^th^ quantile for different levels of incidences, based on these four geographic groups. ArcGIS 9.3 program of Geographical Information System (GIS) was used for spatial analysis. The Kriging method [20, 21] was applied to interpolate each grid cell over a spatial domain. To assess the spatio-temporal relationship among the studied cases in a diffusion map, the distance between the centroid of each grid was calculated as the spatial relationship between the incidence rates of the ILI or EVI cases in each district. We made a surface plot of weekly time series as a gradient interpolated between adjacent weeks and district data points. Distance in the map symbolized relative geographic relationship rather than actual distance.

### Ethics

This study was approved by the Taipei City Hospital Institutional Review Board (IRB) and certified on 24 May, 2012 (Approval Number: TCHIRB-1000905). The process of this reporting also followed the regulation for public health disease surveillance in Taiwan. Our data were fully de-identified and anonymized to protect the participants’ privacy, and only aggregated data were used for further analyses and statistical tests. Thus, the informed consent was not needed in this study.

## Results

### General Epidemiological Characteristics

We first examined the types of infection and sources of reporting through this newly established system. Among the 19,334 syndromic cases reported to the SID-SSS from January 2010 to August 2011, EVI was the most reported syndrome group (77.56%), significantly higher than other major syndrome groups (ILI: 15.75%, red eye: 3.78%, diarrhea: 2.43%, p < 0.001) (). The majority of data were submitted from preschools (42.70%, n = 8256) and primary schools (41.82%, n = 8085), followed by secondary schools (13.88%, n = 2684), and others combined contributing the lowest (1.60%, n = 309).

The overall absenteeism rate for symptomatic school cases was 96.40% (Table 1), particularly higher in preschools and primary schools [97.97% (8088/8256) and 97.70% (7899/8085), respectively] than secondary schools (91.28%, 2450/2684) and other institutions (65.05%, 201/309). The absenteeism rate was higher in cases of EVI (98.79%) and ILI (96.62%), but lower in those with diarrhea (65.74%) and red eye (66.03%) (p<0.001)

**Table 1:** Epidemiological characteristics and sources of reporting of the identified cases of the four syndrome-groups through the SID-SSS in Taipei City, 1 January 2010–31 August, 2011.

Syndrome Groups	Rep. Case No (%)	M/F Ratios	Absenteeism % of Rep.	Cluster %	Family Members w/ S.S. %	Time-Lag from Onset*	Consistency % in Clinical Dx
**EVI**	14995 (77.56%)	1.19	98.79	44.61	16.38	2.6 ± 2.8	97.80
Preschools	7543	1.18	98.75	60.41	12.94	2.5 ± 3.4	97.69
Primary schools	6387	1.14	99.22	27.56	20.42	2.7 ± 2.0	97.84
Secondary schools	958	1.68	96.24	33.72	16.81	3.4 ± 2.2	98.33
Other institutions	107	1.14	99.07	46.73	14.02	2.9 ± 2.1	98.13
**ILI**	3046 (15.75%)	1.17	96.62	19.73	10.64	3.1 ± 8.1	98.88
Preschools	409	0.94	99.02	18.58	19.80	2.7 ± 2.1	99.02
Primary schools	1224	1.16	98.45	18.38	12.42	3.2 ± 10.6	99.35
Secondary schools	1307	1.29	96.10	21.81	6.96	3.1 ± 6.9	98.32
Other institutions	106	1	72.64	14.15	0	2.3 ± 1.8	100.00
**Red Eye**	730 (3.78%)	2.12	66.03	46.44	16.71	2.2 ± 2.2	85.34
Preschools	77	1.33	92.21	20.78	16.88	2.0 ± 1.6	90.91
Primary schools	272	1.41	70.22	46.69	24.63	2.0 ± 1.6	90.44
Secondary schools	303	3.21	72.61	49.17	13.53	2.0 ± 2.5	91.75
Other institutions	78	3.88	0	60.26	1.28	4.0 ± 2.5	37.18
**Diarrhea**	470 (2.43%)	1.04	65.74	79.57	8.30	2.8 ± 1.9	23.19
Preschools	162	1.19	61.11	65.43	16.05	3.0 ± 1.4	20.99
Primary schools	181	1.15	81.22	85.08	3.31	2.2 ± 1.1	12.15
Secondary schools	110	0.83	41.82	90.00	6.36	2.9 ± 2.8	45.45
Other institutions	17	0.42	100.00	88.24	0	6.0 ± 2.0	17.65
**Others**	93 (0.48%)	1.11	96.77	66.67	17.20	3.0 ± 2.1	-
**Total**	19334	1.21	96.40	41.72	15.29	2.7 ± 4.1	95.68

**SID-SSS**: School-based Infectious Disease Syndromic Surveillance System

**EVI**: enterovirus-like illness, **ILI**: influenza-like illness, Reported Case Number (%), Absenteeism % of Reported Cases, Consistency in Clinical Diagnosis (Dx), Family Members with Similar Symptoms (S.S.) %

*Mean ± Standard Deviation (S.D.) of lag days from symptoms onset to data entry

The male-female (M/F) ratio of the syndromic cases was 1.21 (Table 1), much higher than the data (M/F = 1.09) obtained from population-based school children in Taipei. The M/F ratio was significantly higher in red eye syndrome (2.12) (p<0.001).

### School Clusters and Family Members with Similar Symptoms

Cluster events in schools were detected as high as 41.72% among all the reported symptomatic cases (Table 1). Diarrhea was the highest (79.57%) in school clusters whereas red eye (46.44%) and EVI (44.61%) ranked second. Red eye school clusters significantly increased parallel with age (p<0.001), lowest in preschools (20.78%) but more elevated in primary schools (46.69%), secondary schools (49.17%) and other institutions (60.26%). However, EVI school clusters were significantly highest in preschools (60.41%), but lowest in primary schools (27.56%)(p<0.001). ILI clusters accounted for 19.73%, with no differences in various age groups.

In family clusters, 15.29% of the symptomatic school children reported through the SID-SSS had one or more family members with similar symptoms (Table 1). The suspected family clusters for all syndrome groups were high among reported primary school students (18.95%, 1532/8085) and preschool children (13.43%, 1109/8256), but were low in secondary school students (11.18%, 300/2684) and rarely in other institutions (5.18%, 16/309), including university students. EVI and red eye syndrome were reported with significant higher percentages in family clusters (16.38% and 16.71%) than that of ILI family clusters (10.64%)(p<0.001). However, ILI family clusters were particularly higher in preschools (19.80%, p < 0.001).

### Spatial Diffusion Patterns of the EVI and ILI Cases

**Fig 2:**
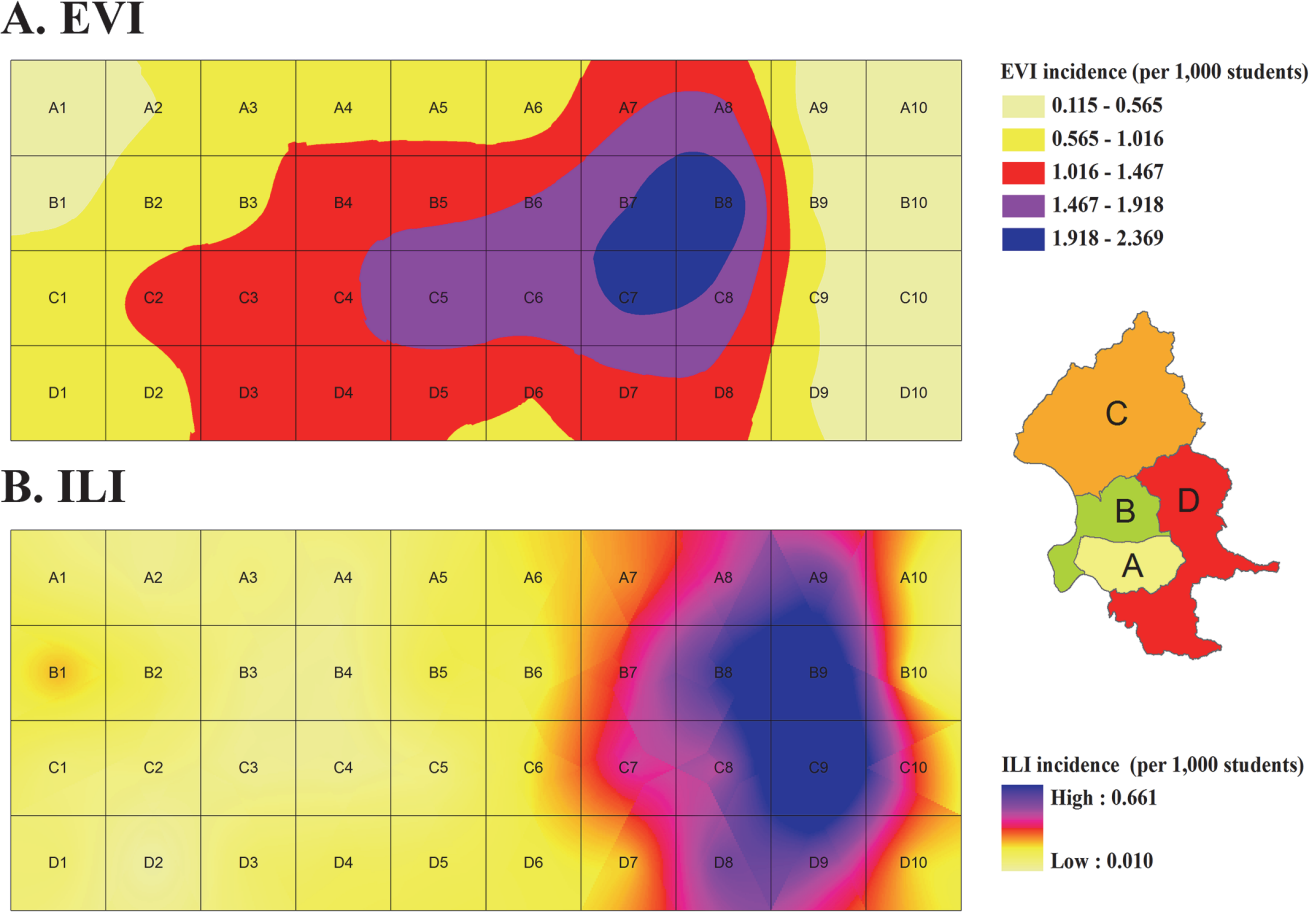
Spatial diffusion of EVI (A) and ILI (B) syndromic cases in the Central Businesses District (CBD) and sub-urban areas of Taipei, at 10 week-peak periods of the epidemic seasons during the study period. (A) EVI diffusion map with mean incidence from Week 19, 2010 to Week 28, 2010 (B) ILI diffusion map with mean incidence from Week 47, 2010 to Week 4, 2011 **The four geographic groups A:** Taipei Central Businesses District (Taipei-CBD), **B**: the para-CBD, **C**: the northern suburbs, and **D**: the south-eastern suburbs.

The diffusion pattern in the incidence rates of EVI cases rose from the northern suburban area C to para-CBD districts B and district D, and finally reached A (Taipei-CBD areas) (Fig 2); there was a three-week interval between these areas and district C. The ILI had a similar diffusion pattern but with a shorter interval. The incidence surged also from C and B, the northern districts of Taipei, and then spread to south-eastern districts D and A (Taipei-CBD areas) (Fig 2).

### Evaluation the Quality of Reporting Syndromic Cases

#### 1. Timeliness

The time-lag periods from onset of the symptoms (based on school teachers’ observations or students’ complaints) to data entry to the SID-SSS were mostly 2–3 days (Table 1). Mean time-lag was significantly shortest in preschools (2.5 ± 3.3), followed by primary schools (2.8 ± 4.5) and secondary schools (3.1 ± 5.1) (p < 0.001). Red eye had the shortest time-lag days (2.2 ± 2.2), but ILI had longer lag days (3.1 ± 8.1)(p< 0.001).

#### 2. Clinical Consistency with Physician Diagnosis

The percentages of consistency between the school personnel’s impression and the doctor’s clinical diagnosis were quite high for cases of EVI (range: 97.69%-98.33%) and ILI (98.32%-100%) in the SID-SSS (Table 1) but this consistency was much lower for diarrhea (12.15%-45.45%). The overall right judgment for the four most prevalent pediatric infectious diseases in Taipei by school staff was 95.68%, particularly high consistency with ILI (98.88%) and EVI (97.80%)(p< 0.001).

#### 3. Temporal Correlation with the Data of the ED-SSS and the LHID2005

**Fig 3:**
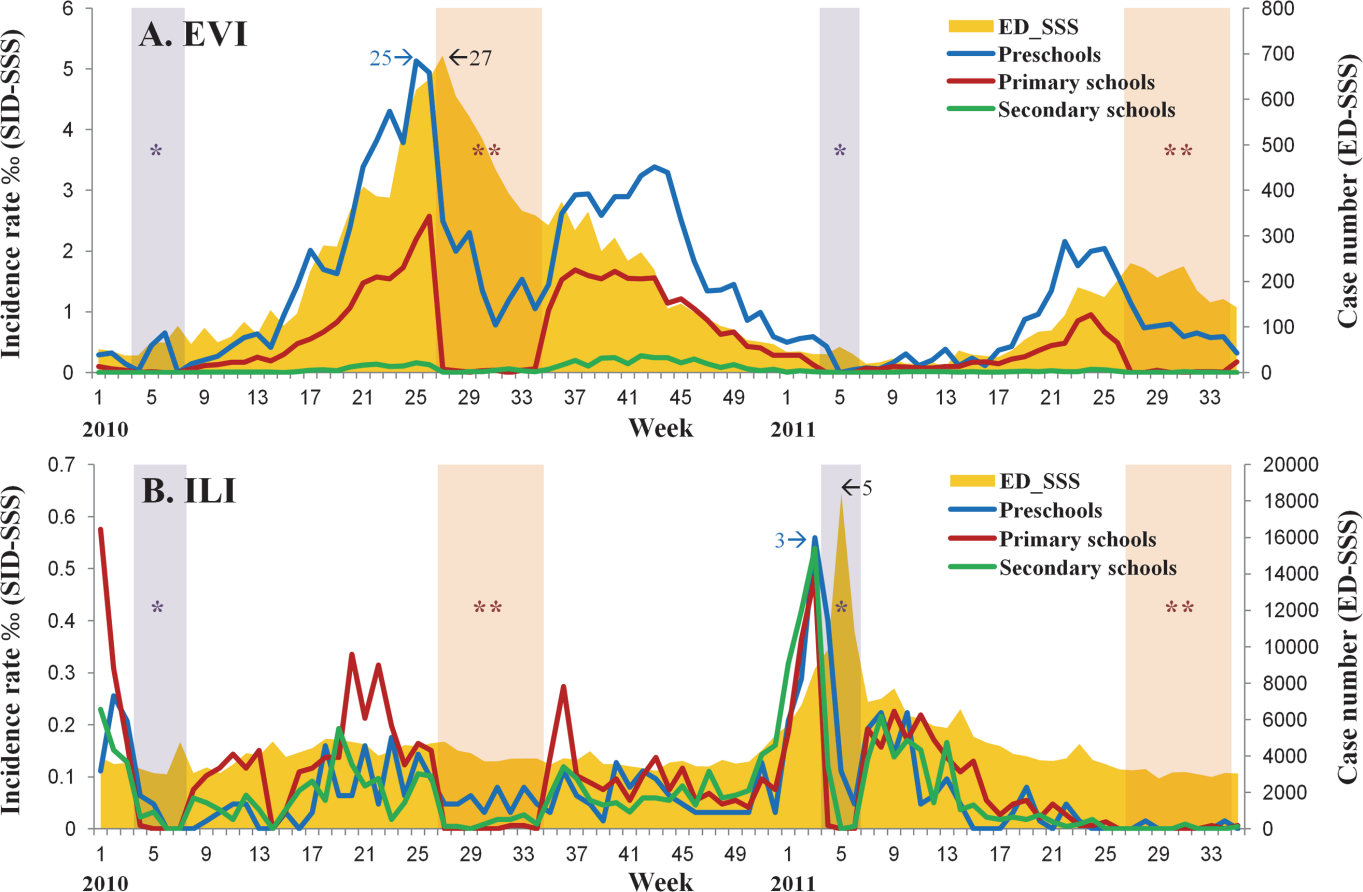
Temporal trends of EVI (A) and ILI (B) cases obtained from the SID-SSS were compared with those collected from the ED-SSS in Taipei City, 1 January, 2010 to 31 August, 2011. **SID-SSS**: School-based Infectious Disease Syndromic Surveillance System **ED-SSS**: Emergency Department-based Syndromic Surveillance System “*” with light purple background indicates the winter vacation (Lunar New Year): Week 4–7, 2010 and 2011. “**” with light pink background indicates the summer vacation: Week 27–34, 2010. Our compiled and summarized data for Fig 3 are freely available in supplementary file in S2 Table.

**Fig 4:**
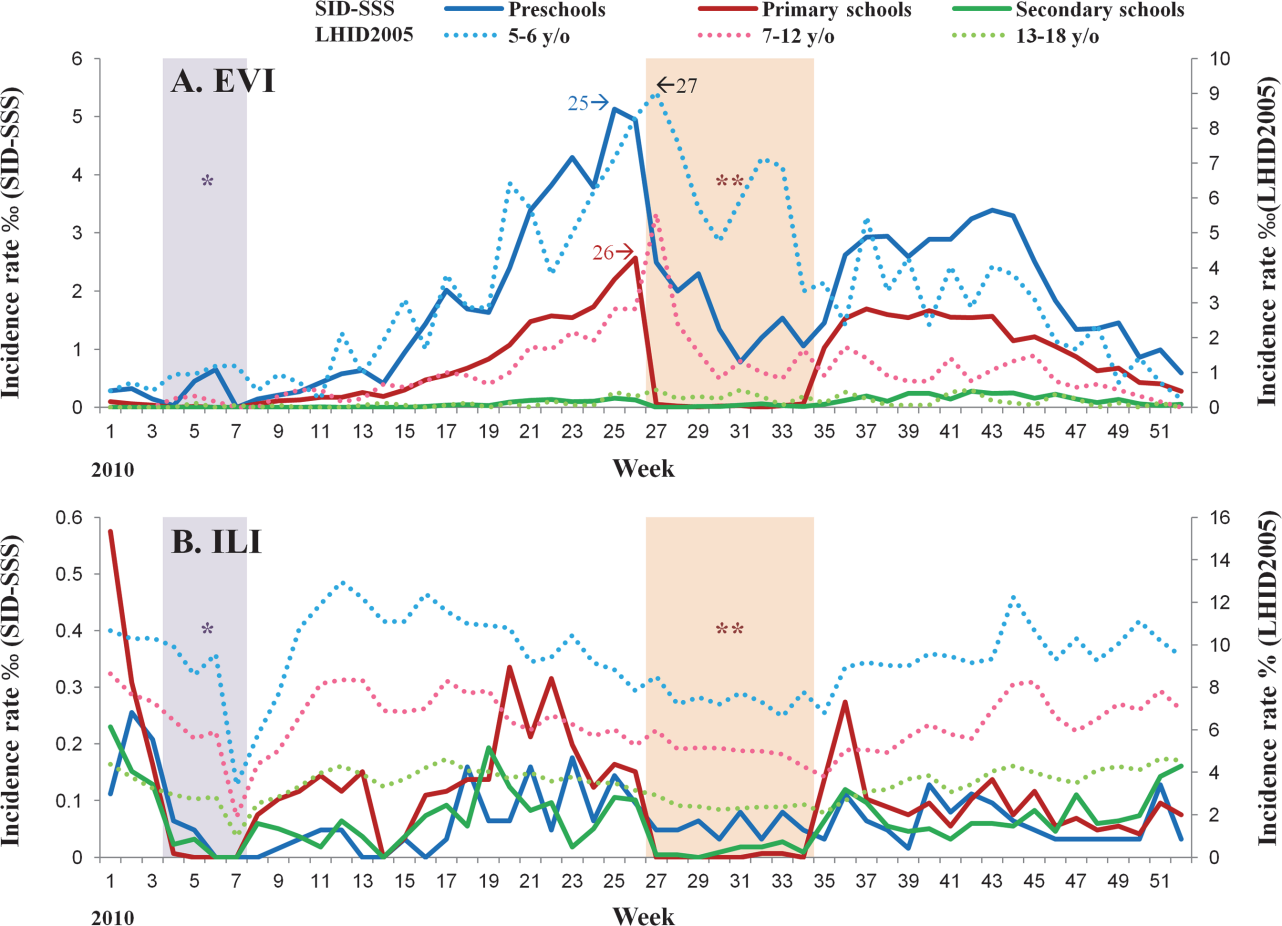
Temporal trends in the incidence rates of EVI (A) and ILI (B) cases among the three age groups in the SID-SSS compared with those collected from the LHID2005 in Taipei City, 1 January 2010 to 31 August 2011. **SID-SSS**: School-based Infectious Disease Syndromic Surveillance System **LHID2005**: Longitudinal Health Insurance Database 2005 “*” with light purple background indicates the winter vacation (Lunar New Year): Week 4–7, 2010 and 2011. “**” with light pink background indicates the summer vacation: Week 27–34, 2010.

The temporal trends in the incidence rates of the four most common syndrome groups of diseases reported to the SID-SSS during the study period were further compared with the counts of syndromic cases reported to the ED-SSS (Fig 3, S1 Fig). In addition, age-group specific trends in the incidence of the EVI and ILI cases in the SID-SSS were further compared with those data of the same age groups from the LHID2005, which provide patients’ information without interference from school breaks (Fig 4).

**EVI:** The trend of EVI cases in the SID-SSS had two waves within a year, one in the spring and the other in the autumn. This pattern was parallel with the ED-SSS (shown as high correlation between the two systems, Table 2) (Fig 3A).

**Table 2:** Pearson’s correlation coefficients of the four syndrome groups between the SID-SSS and the ED-SSS in Taipei City, 1 January, 2010 to 31 August, 2011.

Correlation.	Semester 1	Semester 2	Semester 3	LNY 2010	SMV 2010	LNY 2011	SMV 2011
**EVI**	0.976**	0.818**	0.941**	-0.218	0.796*	-0.579	0.538
**ILI**	0.410	0.954**	0.887**	-0.494	-0.360	-0.540	0.215
**Red eye**	0.598**	0.379	-0.310	NA	0.914**	-0.603	0.717*
**Diarrhea**	0.159	-0.239	0.196	-0.451	-0.041	N.A.	0.048

**SID-SSS**: School-based Infectious Disease Syndromic Surveillance System

**ED-SSS**: Emergency Department-based Syndromic Surveillance System

**Correlation**: Pearson’s correlation coefficient

**EVI:** enterovirus-like illness; **ILI**: influenza-like illness

Semester 1 (Week 8–26, 2010) Semester 2 (Week 35, 2010-Week 3, 2011) Semester 3 (Week 7–26, 2011)

**LNY**: Lunar New Year, **SMV**: Summer Vacation

**. Correlation was significant at the 0.01 level (2-tailed).

*. Correlation was significant at the 0.05 level (2-tailed).

**N.A.:** Cannot be computed since none of the variables is constant in the SID-SSS.

Three age distributions of EVI cases in the SID-SSS revealed clear pattern variations, with the highest incidence among preschool children, followed by primary school children. To evaluate the timeliness to detect the first EVI-peak from the three data sets, we found that preschool children in the SID-SSS appeared the earliest in Week 25 (one week prior to summer vacation) (Fig 3A), that was two weeks earlier than those identified in Week 27 from both the ED-SSS and 5-6-year olds in the LHID2005. The correlation of the EVI cases between the SID-SSS and the ED-SSS was high through Semester 1, 2 and 3 (Table 2), but low during the Lunar New Year holidays and summer vacation periods. Compared to the LHID2005 (S1 Table), the correlation coefficients were higher in preschool and primary school children.

**ILI:** The SID-SSS detected three ILI peaks in 2010, one in January and the others before and after the summer vacation, with similar patterns as the ED-SSS, except for summer and Lunar New Year vacations (Fig 3B). Another ILI peak in January 2011 (late of Semester 2) was detected two weeks earlier than the one identified in the ED-SSS, with a high correlation between the two systems [Pearson correlation = 0.95, p <0.01] (Table 2).

Although primary school children had the highest incidence rate of ILI in the SID-SSS, the ILI in the three age groups (preschool, primary and secondary school children) all peaked at Week 3, 2011 before the winter break. This was also presented two weeks earlier than Week 5 in the ED-SSS (Fig 4B). However, the correlation was not significant when comparing age-group-specific trends in ILI cases in the SID-SSS with those in the LHID2005 (S1 Table).

**Red eye:** Red eye involved several peaks during the periods of pre- and post- summer vacation (S1A Fig), presenting a similar trend as EVI. The incidence rates of this syndrome were higher among children of primary and secondary schools but lower than those in preschools (p < 0.001) (S1A Fig).

**Diarrhea:** Diarrhea cases were reported at a relatively low frequency, with sporadic peaks presented in spring and autumn. No similarity in trends of diarrhea was identified among the four different age groups (S1B Fig).

In summary, the pre-diagnostic data reported from the SID-SSS offered a two-week advance in detecting the peaks of EVI and ILI. During the three semesters, the Pearson’s correlation coefficient was the highest in EVI (ranged 0.818–0.976) than those of the other three syndrome groups (Table 2). Even though the trends of ILI did not show compatible correlation at the beginning, the Pearson’s correlation coefficient reached 0.95 in the second semester and remained high in the third semester after the training program. Therefore, the temporal trends of these two diseases with the SID-SSS reflect the actual situation in the community, as they are verified by the parallel data patterns from the ED-SSS with significant correlations during the school semesters from 2010 to 2011. This is supported by the results of high percentages of consistency between the initial pre-diagnoses judged and reported by school personnel and the physicians’ clinical diagnoses, up to 97.80% in EVI and 98.88% in ILI.

#### 4. Practical Concerns in Application of the SID-SSS in Public Health

The SID-SSS in Taipei is a cost-effective electronic system. Fees spent on traditional facsimile communication were US$13,200 annually. In 2009, consumables including paper and ink cost US$2,772. The total operation costs on equipment and supplies decreased 95.5% (from US$15,972 in 2009 to US$720 in 2010). Time invested from data recruitment to analysis was 1,788 hours per month on average under the paper-format system, but reduced to 720 hours per month with the SID-SSS. There had been a 120-minute gap from data processing to the commencement of infection controls using traditional reporting, but such a gap was shortened to only 20 minutes with the SID-SSS.

## Discussion

This novel syndromic surveillance system with its high representativeness for 0-18-year-olds in Taipei schools is able to detect timely signals for common pediatric infectious diseases. There are four major strengths in the SID-SSS. First, daily report of pre-diagnosis data from bottom-up non-medical professionals in schools detected earlier peaks of EVI and ILI cases than those found from the conventional hospital-based surveillance system. School teachers and nurses participated in public health activities and shared the workload with local public health professionals, thus minimizing the size of clusters and the scale of epidemics. Second, inter-departmental coordination by the three government agencies in health, education, and social welfare through top-down systematic approaches helped to reach ultimate common goals of public health. Third, applying computer technology to infectious disease surveillance provided user-friendly interface that improved efficiency and reduced the cost. Fourth, an integrated data analysis and the follow-up spatial-temporal epidemiological information may help to identify high risk populations and areas for public health intervention [22]. Therefore, the SID-SSS can guide public health professionals in using evidence-based data to implement more effective prevention and control measures.

Surveillance of infectious diseases in schools frequently serves as the first-line alert at the beginning stage of epidemics [23]. Syndromic surveillance is especially useful to detect unexpected events at the earliest possible time [13, 24]. For influenza with its short incubation period and rapid spread, school absenteeism that has been used for the timely monitoring of ILI cases in communities [25–28] frequently had low specificity due to false alarms by reasons other than illness [28]. Cases with the SID-SSS have better specificity because the sick leave requests were reported only if their absenteeism was associated with infectious diseases. In addition, the SID-SSS is also very timely and sensitive in identifying mild cases, particularly the most sensitive age groups at the earliest time. Teachers meeting with students every day were able to observe the flare-up symptoms and signs in advance of medical professionals.

The pre-diagnostic data reported from the SID-SSS offered a 1-2-week advance in detecting the peaks of EVI and ILI, indicating a marked improvement from the hospital-based surveillance system. The time difference between epidemic peaks with the SID-SSS and the ED-SSS can be explained by their coverage of different age groups [29] and health-seeking behaviors. The ED-SSS at the selected medical centers offers the medical information all-year-round, across all-age-groups and most of severe cases, whereas the SID-SSS targeting mostly at mild cases among school-age-children offers a daily reliable data from the community-based, pre-diagnosis surveillance. Therefore, the SID-SSS cannot completely replace the ED-SSS but these two systems can complement each other. Importantly, the EVI cases increased in preschool children as the first peak in the SID-SSS not only provided the earliest warning signal as the trumpet call for a potentially emerging enterovirus epidemic but also reflected the true EVI-trends in local communities by complementing the schools’ break periods. On the contrary, ILI cases of preschools, primary, and secondary schools all peaked in the same week, implying the fast antigenic drift of influenza viruses can undergo a rapid spread in all age groups. Therefore, a future vaccination for EV71 (the most important causing agent for severe EVI) should target preschool children [6, 30], as was polio vaccine [31], whereas vaccination of influenza, particularly for novel influenza viruses with pandemic potential, needs to cover both preschool and older school children to quickly reduce the numbers of susceptible children.

The EVI and ILI cases had similar diffusion patterns within Taipei City, both from the northern suburbs toward Taipei CBD (the national political and economic center with the highest density of medical centers and clinics). The northern suburban area C, bordering Keelung County, had a similar outbreak of acute hemorrhagic conjunctivitis caused by the Coxsackie virus A24 which occurred in 2007 [21]. Having a similar diffusion pattern, however, the spread of ILI was faster than EVI. With higher parental social-economic level, the Taipei CBD was more protected from infectious diseases than suburban districts, implying the importance to increase public health prevention measures for both of these two important pediatric infectious diseases in suburban areas.

The SID-SSS may help to estimate the diseases transmissibility caused by different strains within the same type/subtype/genotype of enteroviruses or influenza viruses through identifying the secondary cases at schools (school clusters) and in families (family clusters). The risk factors of pediatric infectious diseases are multi-factorial. Higher male to female ratios of EVI and red eye cases in the SID-SSS, similar to previous findings [32], may be attributed to gender-related behaviors. This implies that hygiene education should be targeted more towards boys. Household transmission of ILI cases showed a higher rate among younger children who may have had more frequent body contact with family members [33] or virus-contaminated fomites [34]. Hence, the way to improve hygiene for younger-age children is important for parents and child-care personnel. Voluntary household-based quarantine or external isolation should be encouraged to limit viruses from spreading [35].

One of the major strengths of the SID-SSS is the highest quality of coverage and representativeness through the integration framework among three departments of Taipei City government, namely, health, education and welfare. Moreover, the SID-SSS is not only a reporting system but also a documentation system for all follow-up actions involving a broad spectrum of services offered from case identification to recording follow-up prevention and control measures. School administrators can also access the surveillance data at the same time as the officials of these three government bureaus on a real-time basis. In addition, this internet-accessible reporting system has better acceptability than paper-reporting system due to a more user-friendly website interface. What is perhaps more impressive is that local school teachers/nurses become important implementers, providing essential public health intervention measures such as isolation, seasonal flu vaccination, school closure, and class suspension. Furthermore, our data showed that the reporting of EVI surged and correlation of ILI improved in the second semester of 2010, after several training workshops aimed at the public health significance of surveillance and usefulness of the SID-SSS were held. Again, all these results illustrate the significance to evaluate the strengths and weaknesses of this newly developed surveillance system.

Our evaluation of the SID-SSS operating for the first 20 months, however, identified several limitations. First, the data were limited by holidays and school breaks, particularly the data from Lunar New Year holidays and the flu season remained bottle-necked. Second, the specificity of SID-SSS requires improvement by taking specimens via proportional sampling for better integration with virological surveillance, similar to many sentinel-physician influenza surveillance systems [36–38]. Third, inequality of reporting was noted among different disease syndrome-groups, and with lower reporting from colleges, universities and after-school centers. The institutions with shortage of personnel responsible for reporting, and the colleges/universities protecting academic freedom may result in lower proportion of reporting. Interactive mode with two-way communications in the web 2.0 version could be helpful to remove these barriers and thus reaching the mutual goal of public health. Fourth, the follow-up case management information is recorded to the SID-SSS with an optional text format blank to be filled in (rather than a standard format), including transfers to hospital care. However, the proportion of cases visited EDs or outpatient clinics cannot be accurately measured. Future improvement needs to design user-friendly options to track the follow-up medical care and control measures in more detail.

Schools are the best place for health control measures (i.e. hygiene behavior) and ideal training sites for prevention measures. To enhance the efficiency and effectiveness of reporting, we suggest giving “School of the Month” awards for recognizing the most dedication in reporting, along with continuing training programs, and simple pre- and post-training feedback are necessary. In addition, incorporating valuable information, including the subsequent medical transfers, vaccination records, child’s growth development progress, body mass index, asthma and history of chronic diseases [39–41] into the SID-SSS can maximize its usefulness. However, the surveillance of school children should adhere to safeguards to minimize stigmatizing or labeling [42].

For countries with limited medical resource, a school-based syndromic surveillance system to quickly detect pediatric cases can be cost-effective. The SID-SSS initially piloted on 17 November, 2009 in Taipei City and extended to the neighboring New Taipei City since 30 December, 2010, and applied to Taitung County since 17 September, 2014 and Taoyuan County since 1 October, 2014. Besides geographical networking and collaboration with academic institutions, more evidence-based strategies can be developed and adequately evaluated to facilitate child health and social security. A novel surveillance through social media [43] could be added to complement the weaknesses in lower reporting in colleges and universities. As more and more humans are infected with novel avian influenza viruses [44–48], our goal is to provide an alternative system to detect potentially pandemic threats from emerging infectious diseases of unknown etiological agents, particularly in rural areas near poultry farms where many zoonotic infections originated.

### Data source

The original databank is established and provided by Department of Health, Taipei City Government, Taiwan, Republic of China (R.O.C.).

## Supporting Information

S1 Fig Temporal trends of red eye (A) and diarrhea (B) cases obtained from the SID-SSS were compared with those from the ED-SSS in Taipei City, 1 January, 2010 to 31 August, 2011. **SID-SSS**: School-based Infectious Disease Syndromic Surveillance System **ED-SSS**: Emergency Department-based Syndromic Surveillance System “*” with light purple background indicates the winter vacation (Lunar New Year): Week 4–7, 2010 and 2011. “**” with light pink background indicates the summer vacation: Week 27–34, 2010.
(DOCX)

S1 Table Temporal correlation of EVI and ILI cases detected from the SID-SSS and the LHID2005 among different levels of schools and during study periods. **SID-SSS**: School-based Infectious Disease Syndromic Surveillance System **LHID2005**: Longitudinal Health Insurance Database 2005**Correlation.:** Pearson’s correlation coefficient **EVI**: enterovirus-like illness; **ILI**: influenza-like illness **LNY**: Lunar New Year, **SMV**: Summer Vacation # Trends in preschools of the SID-SSS were compared with year 5–6 age-group of the LHID2005 ** Correlation was significant at the 0.01 level (2-tailed). * Correlation was significant at the 0.05 level (2-tailed). N.A.: Cannot be computed since none of the variables is constant in the SID-SSS.
(DOCX)

S2 Table The compiled and summarized data for Fig 3: The incidence rates of EVI and ILI cases per 1000 person in the SID-SSS and the total numbers of EVI and ILI cases in the ED-SSS, January 1, 2010- August 31, 2011, Taipei, Taiwan. The data are available upon request, and readers of *PLoS One* may contact the corresponding author, Dr. Muh-Yong Yen. Contact information: **Dr. Muh-Yong Yen,** Kunming Branch, Dept. of Medicine, Taipei City Hospital, 100 Kunming Street, 4^th^ floor, Taipei, Taiwan (10844), Republic of China (R.O.C.), **Tel**: **+**886–921699678, **E-mail**: myyen1121@gmail.com
(DOCX)
