# Supplementary material for: Early Detection for Cases of Enterovirus- and Influenza-Like Illness through a Newly Established School-Based Syndromic Surveillance System in Taipei, January 2010 ~ August 2011
Source: PLoS One. 2015 Apr 15;10(4):e0122865. doi: 10.1371/journal.pone.0122865 (PMC4398411; doi:10.1371/journal.pone.0122865)
Supplement: S1 Fig — SID-SSS: School-based Infectious Disease Syndromic Surveillance System ED-SSS: Emergency Department-based Syndromic Surveillance System “*” with light purple background indicates the winter vacation (Lunar New Year): Week 4–7, 2010 and 2011. “**” with light pink background indicates the summer vacation: Week 27–34, 2010. (DOCX) [file pone.0122865.s001.docx]

**Figure S1. Temporal trends of red eye (A) and diarrhea (B) cases obtained from the SID-SSS were compared with those from the ED-SSS in Taipei City, 1 January, 2010 to 31 August, 2011.**


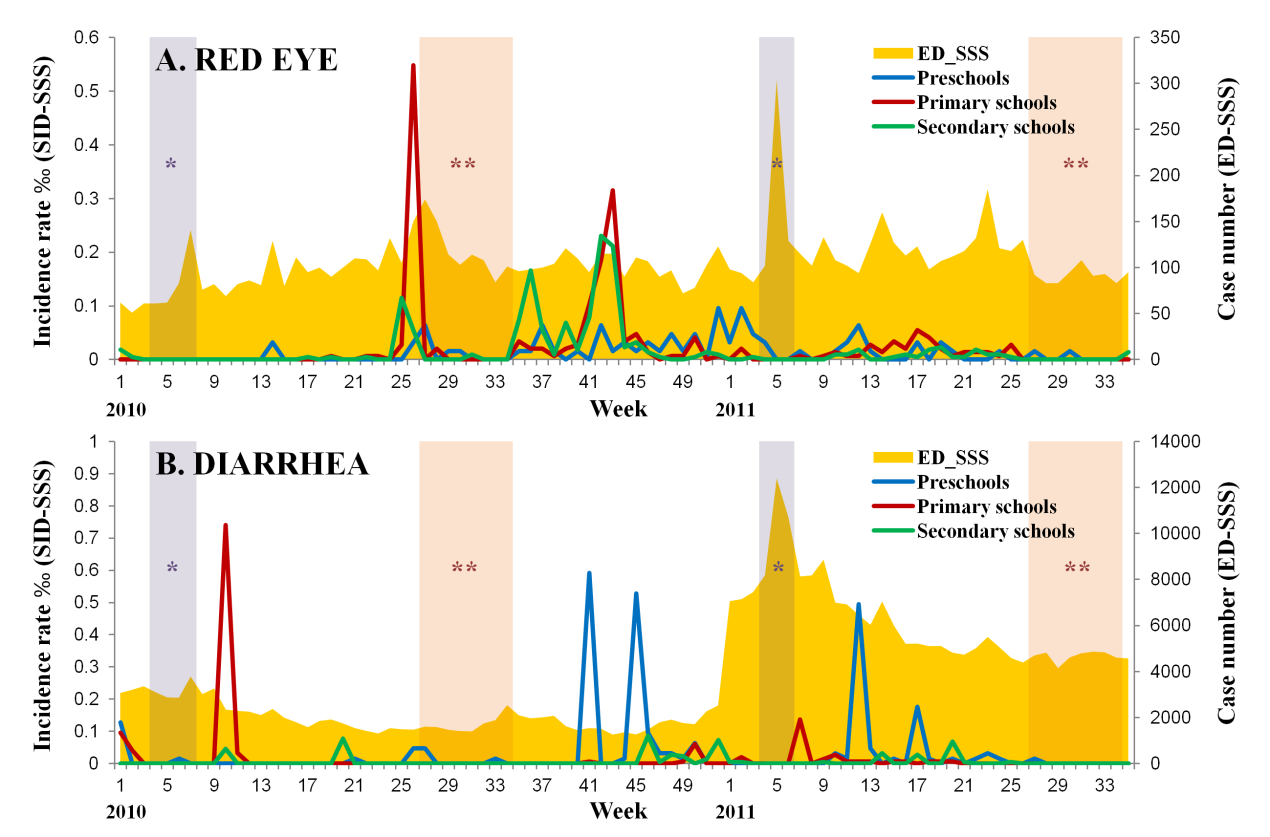


“*” with light purple background indicates the winter vacation (Lunar New Year): Week 4-7, 2010 and 2011.

“**” with light pink background indicates the summer vacation: Week 27-34, 2010.

**SID-SSS**: School-based Infectious Disease Syndromic Surveillance System

**ED-SSS**: Emergency Department-based Syndromic Surveillance System
