# Supplementary material for: Early Detection for Cases of Enterovirus- and Influenza-Like Illness through a Newly Established School-Based Syndromic Surveillance System in Taipei, January 2010 ~ August 2011
Source: PLoS One. 2015 Apr 15;10(4):e0122865. doi: 10.1371/journal.pone.0122865 (PMC4398411; doi:10.1371/journal.pone.0122865)
Supplement: S1 Table — SID-SSS: School-based Infectious Disease Syndromic Surveillance System LHID2005: Longitudinal Health Insurance Database 2005Correlation.: Pearson’s correlation coefficient EVI: enterovirus-like illness; ILI: influenza-like illness LNY: Lunar New Year, SMV: Summer Vacation # Trends in preschools of the SID-SSS were compared with year 5–6 age-group of the LHID2005 ** Correlation was significant at the 0.01 level (2-tailed). * Correlation was significant at the 0.05 level (2-tailed). N.A.: Cannot be computed since none of the variables is constant in the SID-SSS. (DOCX) [file pone.0122865.s002.docx]

**Table S1. Temporal correlation of EVI and ILI cases detected from the SID-SSS and the LHID2005 among different levels of schools and during study periods.**

| **Correlation.** | | **Enterovirus-like Illness (EVI)** | | | **Influenza-like Illness (ILI)** | | |
| --- | --- | --- | --- | --- | --- | --- | --- |
|  |  | **Different Levels of Schools** | | | | | |
| **Periods** | **Weeks** | **Preschools#** | **Primary**  **Schools** | **Secondary**  **Schools** | **Preschools#** | **Primary**  **Schools** | **Secondary**  **Schools** |
| **LNY** | W4-7 | 0.16 | 0.68 | N.A. | 0.56 | 0.48 | 0.54 |
| **Semester 1** | W8-26 | 0.91**^**^** | 0.97**^**^** | 0.68**^**^** | -0.13 | -0.03 | 0.18 |
| **SMV** | W27-34 | 0.59 | 0.49 | -0.02 | -0.15 | -0.2 | -0.37 |
| **Semester 2** | W35-52 | 0.77**^**^** | 0.73**^**^** | 0.38 | 0.03 | -0.3 | 0.24 |
| **Year 2010** | W1-52 | 0.67^**^ | 0.38^**^ | 0.34^*^ | 0.12 | 0.39^**^ | 0.53^**^ |

**SID-SSS**: School-based Infectious Disease Syndromic Surveillance System

**LHID2005**: Longitudinal Health Insurance Database 2005

**Correlation.:** Pearson’s correlation coefficient

**EVI**: enterovirus-like illness; **ILI**: influenza-like illness

**LNY**: Lunar New Year, **SMV**: Summer Vacation

# Trends in preschools of the SID-SSS were compared with year 5-6 age-group of the LHID2005

**. Correlation was significant at the 0.01 level (2-tailed).

*. Correlation was significant at the 0.05 level (2-tailed).

N.A.: Cannot be computed since none of the variables is constant in the SID-SSS.
