# Supplementary material for: Early Detection for Cases of Enterovirus- and Influenza-Like Illness through a Newly Established School-Based Syndromic Surveillance System in Taipei, January 2010 ~ August 2011
Source: PLoS One. 2015 Apr 15;10(4):e0122865. doi: 10.1371/journal.pone.0122865 (PMC4398411; doi:10.1371/journal.pone.0122865)
Supplement: S2 Table — The data are available upon request, and readers of PLoS One may contact the corresponding author, Dr. Muh-Yong Yen. Contact information: Dr. Muh-Yong Yen, Kunming Branch, Dept. of Medicine, Taipei City Hospital, 100 Kunming Street, 4th floor, Taipei, Taiwan (10844), Republic of China (R.O.C.), Tel: +886–921699678, E-mail: myyen1121@gmail.com (DOCX) [file pone.0122865.s003.docx]

**Table S2. The compiled and summarized data for Figure 3: The incidence rates of EVI and ILI cases per 1000 person in the SID-SSS and the total numbers of EVI and ILI cases in the ED-SSS, January 1, 2010- August 31, 2011, Taipei, Taiwan.**

| Year | Week | SID-SSS (EVI) | | | ED-SSS  (EVI) | SID-SSS (ILI) | | | ED-SSS  (ILI) |
| --- | --- | --- | --- | --- | --- | --- | --- | --- | --- |
|  |  | **Preschools** | **Primary**  **Schools** | **Secondary**  **Schools** |  | **Preschools** | **Primary**  **Schools** | **Secondary**  **Schools** |  |
| 2010 | 1 | 0.287701 | 0.095936 | 0 | 51 | 0.111884 | 0.575618 | 0.230188 | 3914 |
| 2010 | 2 | 0.319668 | 0.061673 | 0 | 47 | 0.255734 | 0.308367 | 0.151924 | 3559 |
| 2010 | 3 | 0.14385 | 0.041116 | 0 | 37 | 0.207784 | 0.164462 | 0.128905 | 3624 |
| 2010 | 4 | 0.031967 | 0 | 0 | 37 | 0.063934 | 0.006853 | 0.023019 | 3296 |
| 2010 | 5 | 0.447535 | 0.013705 | 0 | 67 | 0.04795 | 0 | 0.032226 | 3060 |
| 2010 | 6 | 0.655318 | 0 | 0 | 65 | 0 | 0 | 0 | 3000 |
| 2010 | 7 | 0 | 0 | 0 | 102 | 0 | 0 | 0 | 4773 |
| 2010 | 8 | 0.14385 | 0.061673 | 0 | 62 | 0 | 0.075379 | 0.059849 | 3062 |
| 2010 | 9 | 0.207784 | 0.116494 | 0.004604 | 98 | 0.015983 | 0.102789 | 0.050641 | 3377 |
| 2010 | 10 | 0.271717 | 0.130199 | 0.004604 | 66 | 0.031967 | 0.116494 | 0.03683 | 3068 |
| 2010 | 11 | 0.431551 | 0.171315 | 0.009208 | 78 | 0.04795 | 0.143905 | 0.018415 | 3536 |
| 2010 | 12 | 0.575402 | 0.171315 | 0.004604 | 111 | 0.04795 | 0.116494 | 0.064453 | 4162 |
| 2010 | 13 | 0.639335 | 0.253546 | 0.009208 | 84 | 0 | 0.150757 | 0.03683 | 4039 |
| 2010 | 14 | 0.415568 | 0.191873 | 0.004604 | 137 | 0 | 0 | 0 | 4800 |
| 2010 | 15 | 0.943019 | 0.301514 | 0 | 103 | 0.031967 | 0.034263 | 0.03683 | 3947 |
| 2010 | 16 | 1.438504 | 0.472829 | 0.013811 | 129 | 0 | 0.109642 | 0.07366 | 4198 |
| 2010 | 17 | 2.013906 | 0.555061 | 0.041434 | 222 | 0.031967 | 0.116494 | 0.092075 | 4439 |
| 2010 | 18 | 1.694238 | 0.671555 | 0.046038 | 279 | 0.159834 | 0.137052 | 0.055245 | 4948 |
| 2010 | 19 | 1.630304 | 0.829165 | 0.027623 | 276 | 0.063934 | 0.137052 | 0.193358 | 4893 |
| 2010 | 20 | 2.397507 | 1.069006 | 0.092075 | 355 | 0.063934 | 0.335777 | 0.124301 | 4774 |
| 2010 | 21 | 3.388476 | 1.473309 | 0.124301 | 408 | 0.159834 | 0.212431 | 0.082868 | 4644 |
| 2010 | 22 | 3.820027 | 1.576098 | 0.138113 | 387 | 0.04795 | 0.31522 | 0.096679 | 4021 |
| 2010 | 23 | 4.299528 | 1.541835 | 0.101283 | 384 | 0.175817 | 0.198725 | 0.018415 | 4267 |
| 2010 | 24 | 3.78806 | 1.726855 | 0.105886 | 536 | 0.063934 | 0.123347 | 0.050641 | 4607 |
| 2010 | 25 | 5.130664 | 2.199685 | 0.161131 | 621 | 0.14385 | 0.164462 | 0.105886 | 4524 |
| 2010 | 26 | 4.938864 | 2.569725 | 0.128905 | 645 | 0.0959 | 0.150757 | 0.101283 | 4706 |
| 2010 | 27 | 2.493407 | 0.054821 | 0.004604 | 696 | 0.04795 | 0 | 0.004604 | 4788 |
| 2010 | 28 | 1.997922 | 0.02741 | 0 | 607 | 0.04795 | 0 | 0.004604 | 4296 |
| 2010 | 29 | 2.301606 | 0.006853 | 0.018415 | 563 | 0.063934 | 0 | 0 | 4155 |
| 2010 | 30 | 1.342604 | 0.02741 | 0.018415 | 513 | 0.031967 | 0 | 0.009208 | 3712 |
| 2010 | 31 | 0.783185 | 0.034263 | 0.03683 | 446 | 0.079917 | 0 | 0.018415 | 3713 |
| 2010 | 32 | 1.198753 | 0 | 0.059849 | 393 | 0.031967 | 0.006853 | 0.018415 | 3854 |
| 2010 | 33 | 1.534404 | 0.034263 | 0.027623 | 355 | 0.079917 | 0.006853 | 0.027623 | 3865 |
| 2010 | 34 | 1.054903 | 0.061673 | 0.013811 | 345 | 0.04795 | 0 | 0.009208 | 3863 |
| 2010 | 35 | 1.454487 | 1.02789 | 0.055245 | 323 | 0.031967 | 0.137052 | 0.064453 | 3528 |
| 2010 | 36 | 2.621274 | 1.52813 | 0.119698 | 375 | 0.111884 | 0.274104 | 0.119698 | 3883 |
| 2010 | 37 | 2.924958 | 1.692592 | 0.197961 | 312 | 0.063934 | 0.102789 | 0.096679 | 3783 |
| 2010 | 38 | 2.940941 | 1.596656 | 0.105886 | 353 | 0.04795 | 0.089084 | 0.055245 | 4264 |
| 2010 | 39 | 2.589307 | 1.541835 | 0.239395 | 266 | 0.015983 | 0.075379 | 0.046038 | 3556 |
| 2010 | 40 | 2.892991 | 1.665182 | 0.243999 | 296 | 0.127867 | 0.095936 | 0.050641 | 3567 |
| 2010 | 41 | 2.892991 | 1.548688 | 0.142716 | 245 | 0.079917 | 0.054821 | 0.032226 | 3438 |
| 2010 | 42 | 3.244626 | 1.541835 | 0.276225 | 264 | 0.111884 | 0.102789 | 0.059849 | 3335 |
| 2010 | 43 | 3.388476 | 1.562393 | 0.243999 | 226 | 0.0959 | 0.137052 | 0.059849 | 3183 |
| 2010 | 44 | 3.292576 | 1.144384 | 0.248603 | 140 | 0.063934 | 0.075379 | 0.055245 | 3645 |
| 2010 | 45 | 2.50939 | 1.21291 | 0.161131 | 151 | 0.04795 | 0.116494 | 0.082868 | 3738 |
| 2010 | 46 | 1.838088 | 1.048448 | 0.225584 | 132 | 0.031967 | 0.054821 | 0.046038 | 3441 |
| 2010 | 47 | 1.342604 | 0.87028 | 0.142716 | 119 | 0.031967 | 0.068526 | 0.11049 | 3429 |
| 2010 | 48 | 1.358587 | 0.630439 | 0.082868 | 102 | 0.031967 | 0.047968 | 0.059849 | 3790 |
| 2010 | 49 | 1.454487 | 0.671555 | 0.133509 | 94 | 0.031967 | 0.054821 | 0.064453 | 3589 |
| 2010 | 50 | 0.863102 | 0.431714 | 0.064453 | 71 | 0.031967 | 0.041116 | 0.07366 | 3688 |
| 2010 | 51 | 0.990969 | 0.404303 | 0.032226 | 67 | 0.127867 | 0.095936 | 0.142716 | 4332 |
| 2010 | 52 | 0.591385 | 0.280957 | 0.055245 | 62 | 0.031967 | 0.075379 | 0.161131 | 5131 |
| 2011 | 1 | 0.495485 | 0.280957 | 0.009208 | 45 | 0.207784 | 0.18502 | 0.317659 | 5730 |
| 2011 | 2 | 0.559418 | 0.280957 | 0.027623 | 46 | 0.287701 | 0.363188 | 0.418942 | 6826 |
| 2011 | 3 | 0.591385 | 0.130199 | 0.018415 | 40 | 0.559418 | 0.486535 | 0.538639 | 8798 |
| 2011 | 4 | 0.431551 | 0 | 0.009208 | 40 | 0.399584 | 0.006853 | 0.119698 | 9863 |
| 2011 | 5 | 0 | 0 | 0 | 56 | 0.111884 | 0 | 0 | 18382 |
| 2011 | 6 | 0.04795 | 0 | 0 | 40 | 0.04795 | 0 | 0.004604 | 10804 |
| 2011 | 7 | 0.079917 | 0.068526 | 0 | 19 | 0.191801 | 0.191873 | 0.133509 | 6973 |
| 2011 | 8 | 0.063934 | 0.054821 | 0 | 22 | 0.223767 | 0.15761 | 0.216376 | 7141 |
| 2011 | 9 | 0.159834 | 0.095936 | 0.004604 | 30 | 0.14385 | 0.226136 | 0.138113 | 7715 |
| 2011 | 10 | 0.303684 | 0.089084 | 0.013811 | 19 | 0.223767 | 0.171315 | 0.170339 | 6225 |
| 2011 | 11 | 0.111884 | 0.075379 | 0.018415 | 19 | 0.04795 | 0.219283 | 0.151924 | 6038 |
| 2011 | 12 | 0.207784 | 0.075379 | 0.013811 | 22 | 0.063934 | 0.171315 | 0.050641 | 5652 |
| 2011 | 13 | 0.383601 | 0.102789 | 0.013811 | 12 | 0.0959 | 0.137052 | 0.165735 | 5541 |
| 2011 | 14 | 0.111884 | 0.095936 | 0.009208 | 48 | 0.04795 | 0.109642 | 0.03683 | 6574 |
| 2011 | 15 | 0.239751 | 0.171315 | 0.013811 | 39 | 0 | 0.130199 | 0.046038 | 5058 |
| 2011 | 16 | 0.111884 | 0.178168 | 0.009208 | 36 | 0 | 0.054821 | 0.023019 | 4729 |
| 2011 | 17 | 0.367618 | 0.143905 | 0.004604 | 34 | 0 | 0.02741 | 0.018415 | 4553 |
| 2011 | 18 | 0.431551 | 0.219283 | 0.013811 | 48 | 0.031967 | 0.047968 | 0.023019 | 4114 |
| 2011 | 19 | 0.879086 | 0.260399 | 0.023019 | 72 | 0.079917 | 0.054821 | 0.018415 | 3992 |
| 2011 | 20 | 0.959003 | 0.363188 | 0.018415 | 89 | 0.015983 | 0.020558 | 0.027623 | 3769 |
| 2011 | 21 | 1.342604 | 0.445419 | 0.032226 | 92 | 0 | 0.047968 | 0.013811 | 3732 |
| 2011 | 22 | 2.157756 | 0.479682 | 0.018415 | 125 | 0.04795 | 0.02741 | 0.004604 | 3871 |
| 2011 | 23 | 1.758172 | 0.849722 | 0.018415 | 187 | 0.015983 | 0.006853 | 0.009208 | 4668 |
| 2011 | 24 | 1.997922 | 0.952511 | 0.050641 | 177 | 0 | 0.006853 | 0.018415 | 3785 |
| 2011 | 25 | 2.045872 | 0.664702 | 0.046038 | 165 | 0 | 0.013705 | 0 | 3548 |
| 2011 | 26 | 1.614321 | 0.493387 | 0.023019 | 202 | 0 | 0 | 0 | 3272 |
| 2011 | 27 | 1.13482 | 0 | 0 | 240 | 0 | 0 | 0 | 3220 |
| 2011 | 28 | 0.735235 | 0 | 0 | 229 | 0.015983 | 0 | 0 | 3287 |
| 2011 | 29 | 0.767202 | 0.041116 | 0.004604 | 208 | 0 | 0 | 0 | 2767 |
| 2011 | 30 | 0.799169 | 0 | 0 | 222 | 0 | 0 | 0 | 3100 |
| 2011 | 31 | 0.591385 | 0 | 0.013811 | 233 | 0 | 0 | 0.009208 | 3122 |
| 2011 | 32 | 0.655318 | 0.013705 | 0.009208 | 180 | 0 | 0 | 0 | 2997 |
| 2011 | 33 | 0.575402 | 0.013705 | 0.004604 | 154 | 0 | 0.006853 | 0 | 2853 |
| 2011 | 34 | 0.591385 | 0.006853 | 0 | 161 | 0.015983 | 0 | 0 | 3076 |
| 2011 | 35 | 0.319668 | 0.178168 | 0 | 143 | 0 | 0.006853 | 0.004604 | 3041 |

The data are available upon request, and readers of *PLoS One* may contact the corresponding author, Dr. Muh-Yong Yen. Contact information:

**Dr. Muh-Yong Yen,**

Kunming Branch, Dept. of Medicine, Taipei City Hospital, 100 Kunming Street, 4^th^ floor, Taipei, Taiwan (10844), Republic of China (R.O.C.),

**Tel**: **+**886-921699678, **E-mail**: myyen1121@gmail.com
